# Supplementary material for: MicroRNA-146a limits tumorigenic inflammation in colorectal cancer
Source: Nat Commun. 2021 Apr 23;12:2419. doi: 10.1038/s41467-021-22641-y (PMC8065171; doi:10.1038/s41467-021-22641-y)
Supplement: Supplementary file 1 — Supplementary Information [file 41467_2021_22641_MOESM1_ESM.pdf]

Supplementary Information for:

MicroRNA-146a Limits Tumorigenic Inflammation in Colorectal Cancer

Lucien P. Garo[1,2], Amrendra K. Ajay[3], Mai Fujiwara[1], Galina Gabriely[1], Radhika Raheja[1], Chantal Kuhn[1], Brendan Kenyon[1], Nathaniel Skillin[1], Ryoko Kadowaki-Saga[1], Shrishti Saxena[1], and Gopal Murugaiyan[1*]

[1]Ann Romney Center for Neurologic Diseases, Department of Neurology, Brigham and Women's Hospital and Harvard Medical School, Boston, MA 02115, USA.

[2]Boston University School of Medicine, Boston, MA 02118, USA

[3]Renal Division, Department of Medicine, Brigham and Women's Hospital and Harvard Medical School, Boston, MA 02115, USA.

These authors contributed equally: Amrendra K. Ajay and Mai Fujiwara

*Corresponding author: mgopal@rics.bwh.harvard.edu

Supplementary Fig. 1. WT and miR-146a$^{-/-}$ bone marrow chimeras.

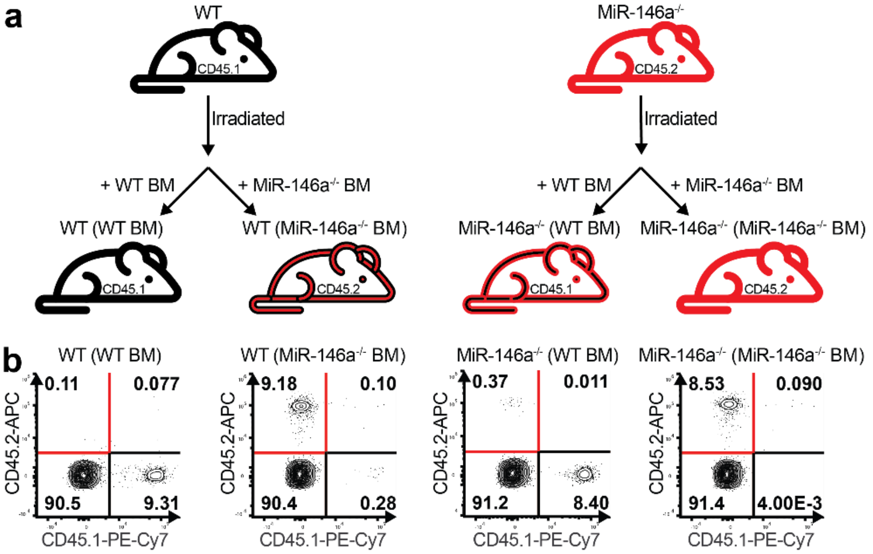

Supplementary Fig. 1. WT and miR-146a$^{-/-}$ bone marrow chimeras.

a, Schematic of WT and miR-146a$^{-/-}$ BM chimeras. CD45.1 WT and CD45.2 miR-146a$^{-/-}$ mice were irradiated and reconstituted with WT or miR-146a$^{-/-}$ 5 x 10^6 bone marrow cells for 6 weeks. Extended license image from stock.adobe.com. **b**, Representative FACS plots of CD45.1 or CD45.2 compartments in these mice after 6 weeks (n = 3). Data representative of ≥2 independent experiments. n = biologically independent replicates per group. Source data are provided as a Source data file.

Supplementary Fig. 2. MiR-146a deletion within myeloid cells promotes CRC development.

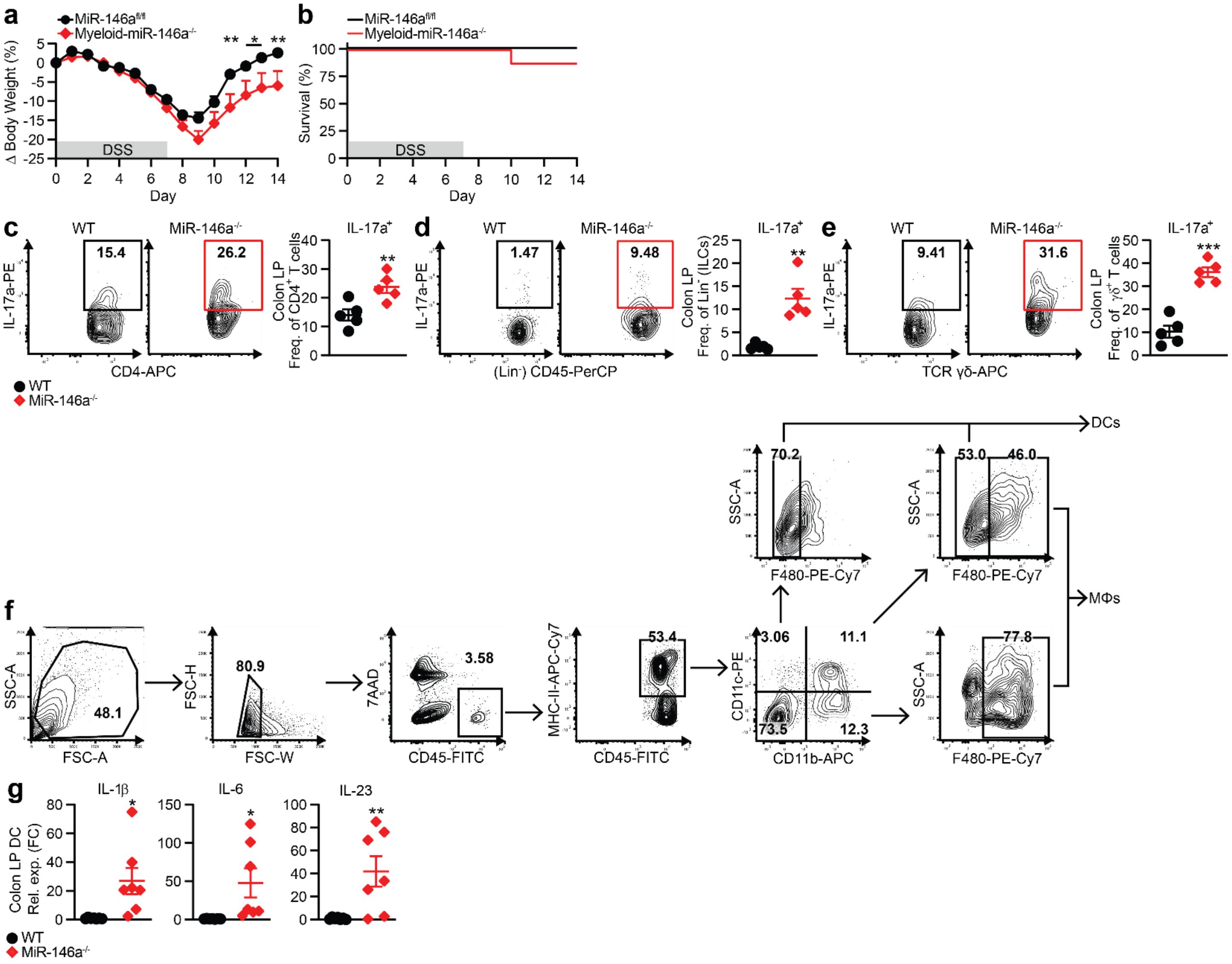

Supplementary Fig. 2. MiR-146a deletion within myeloid cells promotes CRC development.

a,**b** Percent body weight changes (**a**) and survival (**b**) in colitic (3% DSS) miR-146a$^{fl/fl}$ and myeloid-miR-146a$^{-/-}$ mice (n = 8). **c**-**e**, Representative FACS plots and frequencies of IL-17a in CD4^{+} T cells (Th17) (**c**), (Lin^{-}CD45^{+}) ILCs (**d**), and (CD3^{+}γδ^{+}) γδ^{+} T cells (**e**) from the colonic LP of CRC WT and miR-146a$^{-/-}$ mice (n = 5). **f**, Schematic of FACS for LP DCs and MΦs: Total cells → Singlets → Live (7AAD^{-}) CD45^{+} immune cells → MHC-II^{+}CD45^{+} immune cells → CD11c^{+}CD11b$^{+/-}$F480^{-} DCs and CD11c$^{+/-}$CD11b^{+}F480^{+} MΦs. **g**, qPCR of IL-17-inducing cytokines in CD11c^{+} DCs from the colonic LP of WT and miR-146a$^{-/-}$ mice with AOM/DSS-induced CRC (n = 7). qPCR data as FC from WT. Data representative of ≥2 independent experiments. n = biologically independent replicates per group. Mean ± SEM. *p<.05, **p<.01, ***p<.001, by two-way ANOVA with Bonferroni adjustment (**a**) or two-tailed Student's t-test (**c**-**e**,**g**). Source data are provided as a Source data file.

Supplementary Fig 3. Myeloid cell deletion of miR-146a enhances tumorigenic IL-17-promoting cytokines by promoting NOD2-RIPK2 signaling.

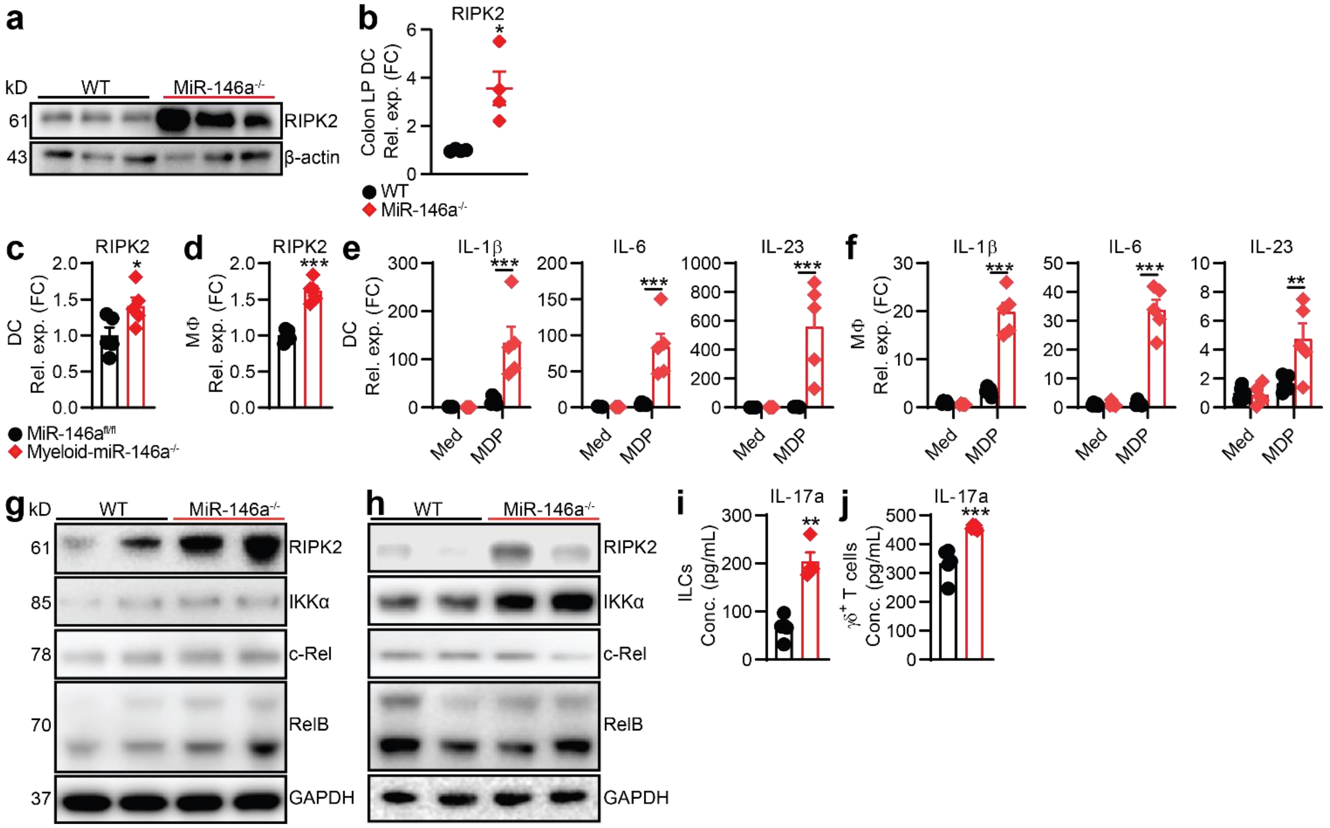

Supplementary Fig 3. Myeloid cell deletion of miR-146a enhances tumorigenic IL-17-promoting cytokines by promoting NOD2-RIPK2 signaling.

a, Western blot of RIPK2 in CRC tissue from WT and miR-146a$^{-/-}$ mice. **b**, qPCR of RIPK2 in CD11c$^+$ DCs from the colonic LP of WT and miR-146a$^{-/-}$ mice with AOM/DSS-induced CRC (n = 4). **c**,**d**, qPCR of RIPK2 in bone marrow-derived DCs (**c**) and MΦs (**d**) from miR-146a$^{fl/fl}$ and myeloid-miR-146a$^{-/-}$ mice stimulated with MDP (10μg/ml) for 24 h (n = 5). **e,f,** qPCR of IL-17-inducing cytokines in miR-146a$^{fl/fl}$ and myeloid-miR-146a$^{-/-}$ BMDCs (**e**) and BMDMs (**f**) stimulated with MDP (10 μg/ml) for 24h (n = 5). **g,h,** Western blots of RIPK2, IKKα, c-Rel, and RelB from WT and miR-146a$^{-/-}$ BMDMs (**g**) and BMDCs (**h**). **i**,**j**, ELISA of IL-17 in ILCs (n =5) (**i**) and γδ$^+$ T cells (n = 4) (**j**) cocultured with WT or miR-146a$^{-/-}$ BMDCs at a 1:1 ILC:DC or γδ$^+$ T cell:DC ratio for 5 days under low-dose Th17-polarizing conditions with TGF-β (0.5 ng/ml), IL-6 (10 ng/ml), and anti-IFN-γ (10 μg/ml). Soluble anti-CD3 and anti-CD28 (1 μg/ml) was added to γδ$^+$T cell:DC cocultures. DCs were prestimulated with MDP for 20h, then washed before coculture. qPCR data as FC from WT (**b**,**i**,**j**), miR-146a$^{fl/fl}$ (**c**,**d**) or miR-146a$^{fl/fl}$ med (**e**,**f**). Data representative of ≥2 independent experiments. n = biologically independent replicates per group. Mean ± SEM. *p<.05, **p<.01, ***p<.001, by two-way ANOVA with Tukey adjustment (**e**,**f**) or two-tailed Student's t-test (**b**-**d**,**i**,**j**). Source data are provided as a Source data file.

Supplementary Fig. 4. MiR-146a deletion within IECs promotes CRC development.

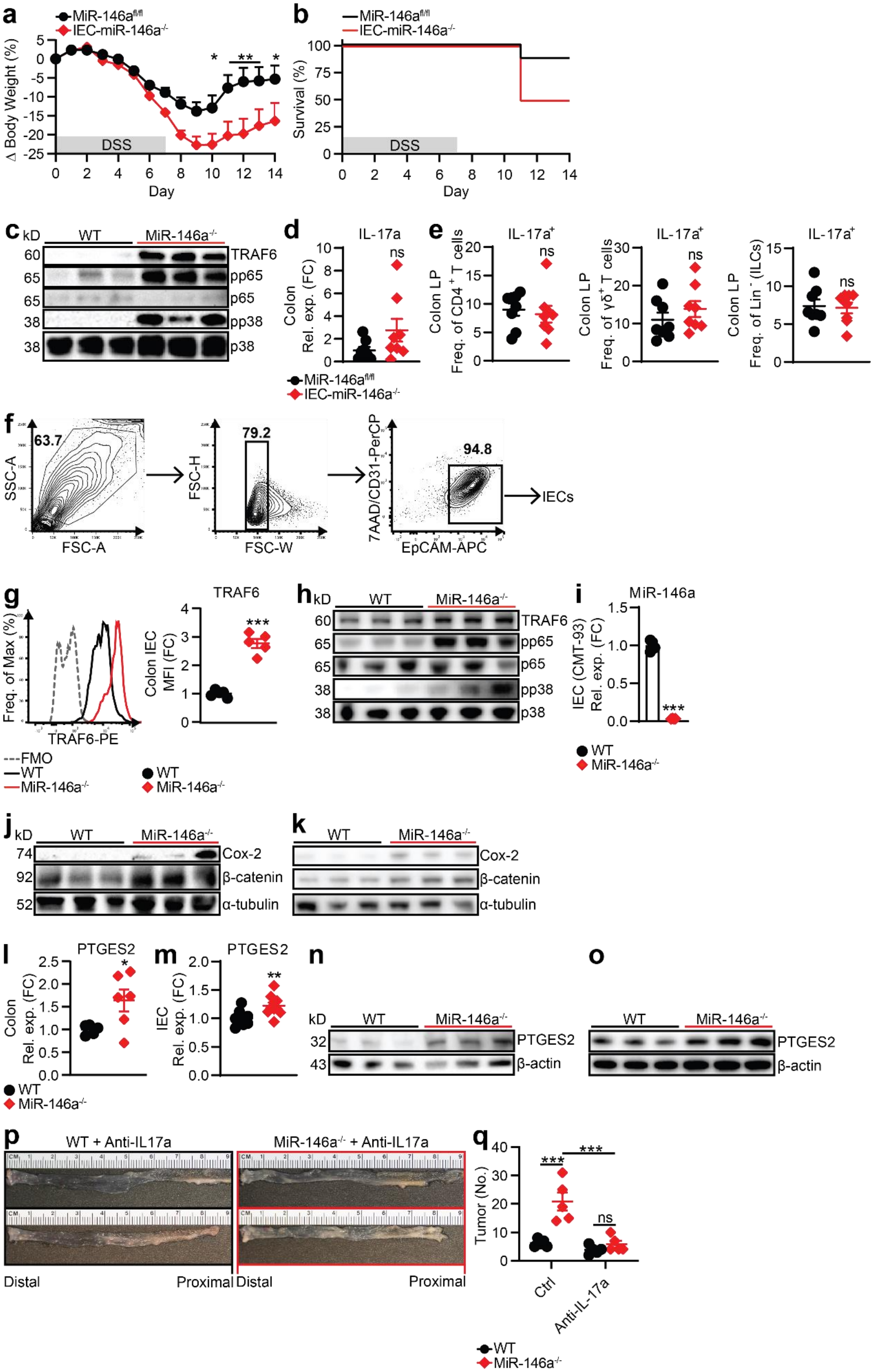

Supplementary Fig. 4. MiR-146a deletion within IECs promotes CRC development.

a,**b**, Percent body weight changes (**a**) and survival (**b**) in colitic (3% DSS) miR-146a$^{fl/fl}$ and IEC-miR-146a$^{-/-}$ mice (n = 8). **c**, Western blots of TRAF6, NF-κB subunit phospho-p65 (pp65), and phospho-p38 (pp38) MAPK in CRC tissue from WT and miR-146a$^{-/-}$ mice. **d**, qPCR of IL-17 in CRC tissue from miR-146a$^{fl/fl}$ and IEC-miR-146a$^{-/-}$ mice with AOM/DSS-induced CRC. qPCR data as FC from miR-146a$^{fl/fl}$ (n = 8) **e**, FACS frequencies of IL-17a in CD4^{+} T cells (Th17), (Lin^{-}CD45^{+}) ILCs, and (CD3^{+}γδ^{+}) γδ^{+} T cell from the colonic LP of CRC miR-146a$^{fl/fl}$ and IEC-miR-146a$^{-/-}$ mice (n = 8). **f**, Schematic of FACS for IECs: Total cells → Singlets → Live (7AAD^{-}) CD31^{-} EpCAM^{+} IECs. **g**, Representatives FACS histograms and MFIs of TRAF6 in FACS-sorted IECs from WT and miR-146a$^{-/-}$ mice with AOM/DSS-induced CRC. MFI as FC from WT (n = 5). **h**, Western blots of TRAF6, pp65, and pp38 in IECs from CRC WT and miR-146a$^{-/-}$ mice. **i**, qPCR of miR-146a in ctrl or miR-146a-silenced CMT-93 IEC line (n = 4). **j**,**k**, Western blots of Cox-2 and β-catenin in CRC tissue (**j**) and IECs (**k**) from CRC WT and miR-146a$^{-/-}$ mice. **l**,**m**, qPCR of PTGES2 in CRC tissue (n = 5-6) (**l**) and IECs (n = 9-10) (**m**) from CRC WT and miR-146a$^{-/-}$ mice. **n**,**o**, Western blot of PTGES2 in CRC tissue (**n**) and IECs (**o**) from CRC WT and miR-146a$^{-/-}$ mice. **p**,**q**, Representative images (**p**) and numbers (**q**) of colonic tumors in WT and miR-146a$^{-/-}$ mice treated with anti-IL-17a (500 μg/mouse) twice a week throughout AOM/DSS CRC induction (n = 5). qPCR data as FC from WT. Data representative of ≥2 independent experiments. n = biologically independent replicates per group (**a**,**b**,**d**,**e**, **g**,**l**,**m**,**q**) or replicates pooled from independent experiments (**i**). Mean ± SEM. *p<.05, **p<.01, ***p<.001, by two-way ANOVA with Bonferroni adjustment (**a**), two-way ANOVA with Tukey adjustment (**q**), or two-tailed Student's t-test (**d**,**e**,**g**,**i**,**l**,**m**). Source data are provided as a Source data file.
