# Supplementary material for: MicroRNA-146a limits tumorigenic inflammation in colorectal cancer
Source: Nat Commun. 2021 Apr 23;12:2419. doi: 10.1038/s41467-021-22641-y (PMC8065171; doi:10.1038/s41467-021-22641-y)
Supplement: Supplementary file 3 — Reporting Summary [file 41467_2021_22641_MOESM3_ESM.pdf]

nature research

Corresponding author(s): Murugaiyan Gopal

Last updated by author(s): March 21, 2021

Reporting Summary

Nature Research wishes to improve the reproducibility of the work that we publish. This form provides structure for consistency and transparency in reporting. For further information on Nature Research policies, see our Editorial Policies and the Editorial Policy Checklist.

Statistics

For all statistical analyses, confirm that the following items are present in the figure legend, table legend, main text, or Methods section.

n/a	Confirmed	
☐	☒	The exact sample size (*n*) for each experimental group/condition, given as a discrete number and unit of measurement
☐	☒	A statement on whether measurements were taken from distinct samples or whether the same sample was measured repeatedly
☐	☒	The statistical test(s) used AND whether they are one- or two-sided<br>*Only common tests should be described solely by name; describe more complex techniques in the Methods section.*
☐	☒	A description of all covariates tested
☐	☒	A description of any assumptions or corrections, such as tests of normality and adjustment for multiple comparisons
☐	☒	A full description of the statistical parameters including central tendency (e.g. means) or other basic estimates (e.g. regression coefficient) AND variation (e.g. standard deviation) or associated estimates of uncertainty (e.g. confidence intervals)
☐	☒	For null hypothesis testing, the test statistic (e.g. *F*, *t*, *r*) with confidence intervals, effect sizes, degrees of freedom and *P* value noted<br>*Give P values as exact values whenever suitable.*
☒	☐	For Bayesian analysis, information on the choice of priors and Markov chain Monte Carlo settings
☒	☐	For hierarchical and complex designs, identification of the appropriate level for tests and full reporting of outcomes
☒	☐	Estimates of effect sizes (e.g. Cohen's *d*, Pearson's *r*), indicating how they were calculated

Our web collection on statistics for biologists contains articles on many of the points above.

Software and code

Policy information about availability of computer code

Data collection	Leica Biosystems Aperio Digital Pathology Slide Scanner was used to acquire microscopy images.<br>BD (Becton Dickinson) Biosciences FACSDiva v8.0.2 was used to acquire flow cytometry data.<br>TargetScan 7.1 and RNAhybrid 2.2 online algorithms were used to acquire microRNA-146a and microRNA-146a mRNA target sequences for binding predictions.<br>Applied Biosystems QuantStudio 7 was used to acquire real-time quantitative PCR data.<br>Western blot images were captured with the Bio-Rad ChemiDoc MP imaging system using Image Lab v1.<br>Fluorescence, absorbance, and luminescence was measured with the GloMax Explorer Multimode Microplate Reader running Glomax Explorer v3.1.0.
Data analysis	BD (Becton Dickinson) Flowjo vs 10.7.1 was used to analyze flow cytometry data and prepare figures.<br>Leica Biosystems Aperio Imagescope 12.4.3 was used to prepare microscopy images.<br>Graphpad Prism 9.0.2 was used to generate figures and to perform statistical analyses.<br>Adobe Illustrator v25.1 was used to arrange figure panels for publication.<br>Microsoft Excel for Office 365 was used to prepare Source Data Files.

For manuscripts utilizing custom algorithms or software that are central to the research but not yet described in published literature, software must be made available to editors and reviewers. We strongly encourage code deposition in a community repository (e.g. GitHub). See the Nature Research guidelines for submitting code & software for further information.

Data

Policy information about availability of data

All manuscripts must include a data availability statement. This statement should provide the following information, where applicable:

- Accession codes, unique identifiers, or web links for publicly available datasets
- A list of figures that have associated raw data
- A description of any restrictions on data availability

The authors declare that data which support the findings in the article and supplementary information are available from the corresponding author upon reasonable request. Source data are provided as a Source Data file.

Field-specific reporting

Please select the one below that is the best fit for your research. If you are not sure, read the appropriate sections before making your selection.

☒ Life sciences ☐ Behavioural & social sciences ☐ Ecological, evolutionary & environmental sciences

For a reference copy of the document with all sections, see nature.com/documents/nr-reporting-summary-flat.pdf

Life sciences study design

All studies must disclose on these points even when the disclosure is negative.

Sample size	Statistical measures were generally not used to predetermine sample size. Sample sizes were based on the experience of investigators from prior experiments in our laboratory and previously published data, as well as the availability of mouse strains and cell counts. Experiments were repeated a minimum of 2-3 times, as noted in figure legends. Sample size n is noted in figure legends. Results were reproducible and statistical significance was apparent.
Data exclusions	Data were not excluded from analysis.
Replication	All replication attempts were successful and consistent with the data presented in the manuscript. The number of replicates are described in each figure legend. Individual data points are shown throughout.
Randomization	Control and experimental mice were assigned randomly into age/sex-matched groups. For in vitro experiments, cells were seeded identically at the onset of the experiments and randomized into the various treatment groups prior to the beginning of experimental protocols.
Blinding	Investigators were not blinded for the majority of the experiments, except for tumor enumeration and evaluation of CRC severity, and for colitis scoring. However, experimental conditions were often evident from the observed phenotypes. There was no risk of bias for in vitro studies from knowing sample details, so blinding was not pertinent. Our experiments were primarily based on flow cytometric and quantitative PCR analyses, which is not subject to the same potential biases as in vivo experiments with more subjective outcomes.

Reporting for specific materials, systems and methods

We require information from authors about some types of materials, experimental systems and methods used in many studies. Here, indicate whether each material, system or method listed is relevant to your study. If you are not sure if a list item applies to your research, read the appropriate section before selecting a response.

Materials & experimental systems

n/a	Involved in the study	
☐	☒	Antibodies
☐	☒	Eukaryotic cell lines
☒	☐	Palaeontology and archaeology
☐	☒	Animals and other organisms
☒	☐	Human research participants
☒	☐	Clinical data
☒	☐	Dual use research of concern

Methods

n/a	Involved in the study	
☒	☐	ChIP-seq
☐	☒	Flow cytometry
☒	☐	MRI-based neuroimaging

Antibodies

Antibodies used	ANTIBODIES USED IN CELL CULTURE<br><br>Purified anti-mouse CD3 Antibody (BioLegend, cat#100202, clone 17A2)<br>https://www.biolegend.com/en-us/products/purified-anti-mouse-cd3-antibody-48?

Purified anti-mouse CD28 Antibody (BioLegend, cat#102102, clone 37.51)
https://www.biolegend.com/en-us/products/purified-anti-mouse-cd28-antibody-117

Ultra-LEAF™ Purified anti-mouse IFN-γ Antibody (BioLegend, cat#505834, clone XMG1.2)
https://www.biolegend.com/en-us/products/ultra-leaf-purified-anti-mouse-ifn-gamma-antibody-7752

ANTIBODIES USED IN FLOW CYTOMETRY

FITC anti-mouse CD3 Antibody (BioLegend, cat# 100204, clone 17A2)
https://www.biolegend.com/en-us/products/fitc-anti-mouse-cd3-antibody-45

APC anti-mouse CD4 Antibody (Biolegend, Cat#100412, clone GK1.5)
https://www.biolegend.com/en-us/products/apc-anti-mouse-cd4-antibody-245

PE anti-mouse CD4 Antibody (BioLegend, Cat#100408, clone GK1.5)
https://www.biolegend.com/en-us/products/pe-anti-mouse-cd4-antibody-250

Brilliant Violet 421™ anti-mouse CD4 Antibody (BioLegend, Cat# 100443, clone GK1.5)
https://www.biolegend.com/en-us/products/brilliant-violet-421-anti-mouse-cd4-antibody-7142

PE anti-mouse IL-17A Antibody (BioLegend, Cat#506904, clone TC11-18H10.1)
https://www.biolegend.com/en-us/products/pe-anti-mouse-il-17a-antibody-1633

APC anti-mouse IL-17A Antibody (BioLegend, Cat#506916, clone TC11-18H10.1)
https://www.biolegend.com/en-us/products/apc-anti-mouse-il-17a-antibody-3540

APC anti-mouse CD11c Antibody (BioLegend, Cat#117310, clone N418)
https://www.biolegend.com/en-us/products/apc-anti-mouse-cd11c-antibody-1813

PE anti-mouse CD11c Antibody (Biolegend, Cat#117308, clone N418)
https://www.biolegend.com/en-us/products/pe-anti-mouse-cd11c-antibody-1816

APC anti-mouse/human CD11b Antibody (Biolegend, Cat#101212, clone M1/70)
https://www.biolegend.com/en-us/products/apc-anti-mouse-human-cd11b-antibody-345

PE/Cyanine7 anti-mouse F4/80 Antibody (Biolegend, Cat#123114, clone BM8)
https://www.biolegend.com/en-us/products/pe-cyanine7-anti-mouse-f4-80-antibody-4070

PE anti-mouse I-A/I-E Antibody (BioLegend, Cat#107608, clone M5/114.15.2)
https://www.biolegend.com/en-us/search-results/pe-anti-mouse-i-a-i-e-antibody-367

APC Anti-mouse TCR γ/δ Antibody (BioLegend, Cat#118116, clone GL3)
https://www.biolegend.com/en-us/products/apc-anti-mouse-tcr-gamma-delta-antibody-6061

PerCP/Cyanine5.5 anti-mouse CD326 (Ep-CAM) Antibody (Biolegend, Cat#118220, clone G8.8)
https://www.biolegend.com/en-us/products/percp-cyanine5-5-anti-mouse-cd326-ep-cam-antibody-5602

EpCAM Mouse mAb (PE Conjugate) (Cell Signaling, Cat#8995, clone VUID9)
https://www.cellsignal.com/products/antibody-conjugates/epcam-vu1d9-mouse-mab-pe-conjugate/8995

APC anti-mouse CD326 (Ep-CAM) Antibody (Biolegend, Cat#118214, clone G8.8)
https://www.biolegend.com/en-us/products/apc-anti-mouse-cd326-ep-cam-antibody-4974

FITC anti-mouse CD45 Antibody (Biolegend, Cat#103108, clone 30-F11)
https://www.biolegend.com/en-us/products/fitc-anti-mouse-cd45-antibody-99

APC anti-mouse CD45 Antibody (Biolegend, Cat#103112, clone 30-F11)
https://www.biolegend.com/en-us/products/apc-anti-mouse-cd45-antibody-97

PerCP anti-mouse CD45 Antibody (Biolegend, Cat#103130, clone 30-F11)
https://www.biolegend.com/en-us/products/percp-anti-mouse-cd45-antibody-4265

PE/Cyanine 7 anti-mouse CD45.1 Antibody (Biolegend, Cat#110730, clone A20)
https://www.biolegend.com/en-us/products/apc-anti-mouse-cd45-1-antibody-2319

APC anti-mouse CD45.2 Antibody (BioLegend, Cat#109830, clone 104)
https://www.biolegend.com/en-us/products/pe-cyanine7-anti-mouse-cd45-2-antibody-4918

Brilliant Violet 421™ anti-mouse Lineage Cocktail (Biolegend, Cat#109814, clone 104)
https://www.biolegend.com/en-us/products/brilliant-violet-421-anti-mouse-lineage-cocktail-7825

PE anti-mouse CD127 (IL-7Rα) Antibody (Biolegend, Cat#135010, clone A7R34)
https://www.biolegend.com/en-us/products/pe-anti-mouse-cd127-il-7ralpha-antibody-6190

PE Mouse Anti-p38 MAPK (pT180/pY182) (BD Biosciences, cat#612565, clone 36/p38 (pT180/pY182)
https://www.bdbiosciences.com/eu/applications/research/b-cell-research/intracellular-antigens/human/pe-mouse-anti-p38-mapk-

pt180py182-36p38-pt180py182/p/612565

PE-conjugated TRAF6 (Abcam, Cat#ab210412, clone 210412)
https://www.abcam.com/pe-traf6-antibody-ep591y-ab210412.html

PE Phospho-NF-κB p65 (Ser536) (93H1) Rabbit mAb (Cell Signaling Technology, Cat#5733, clone 93H1)
https://www.cellsignal.com/products/antibody-conjugates/phospho-nf-kb-p65-ser536-93h1-rabbit-mab-pe-conjugate/5733

PerCP/Cyanine5.5 anti-mouse CD31 Antibody (Biolegend, Cat#102420, clone 390)
https://www.biolegend.com/en-us/products/percp-cyanine5-5-anti-mouse-cd31-antibody-6668

ANTIBODIES USED IN WESTERN BLOTTING

TRAF6 (D21G3) Rabbit mAb (Cell Signaling Technology, Cat#8028S, clone D21G3)
https://www.cellsignal.com/products/primary-antibodies/traf6-d21g3-rabbit-mab/8028

NF-κB p65 (D14E12) XP® Rabbit mAb (Cell Signaling Technology, Cat#8242, clone D14E12)
https://www.cellsignal.com/products/primary-antibodies/nf-kb-p65-d14e12-xp-rabbit-mab/8242

Phospho-NF-κB p65 (Ser536) (93H1) Rabbit mAb (Cell Signaling Technology, Cat#3033, clone 93H1)
https://www.cellsignal.com/products/primary-antibodies/phospho-nf-kb-p65-ser536-93h1-rabbit-mab/3033

p38 MAPK (D13E1) XP® Rabbit mAb (Cell Signaling Technology, Cat#8690, clone D13E1)
https://www.cellsignal.com/products/primary-antibodies/p38-mapk-d13e1-xp-rabbit-mab/8690

Phospho-p38 MAPK (Thr180/Tyr182) (D3F9) XP® Rabbit mAb (Cell Signaling Technology, Cat#4511,clone D3F9)
https://www.cellsignal.com/products/primary-antibodies/phospho-p38-mapk-thr180-tyr182-d3f9-xp-rabbit-mab/4511)

Cox2 (D5H5) XP® Rabbit mAb (Cell Signaling Technology, Cat#12282, clone D5H5)
https://www.cellsignal.com/products/primary-antibodies/cox2-d5h5-xp-rabbit-mab/12282

β-Catenin (D10A8) XP® Rabbit mAb (Cell Signaling Technology, Cat#8480, clone D10A8)
https://www.cellsignal.com/products/primary-antibodies/b-catenin-d10a8-xp-rabbit-mab/8480

Monoclonal Anti-RIPK2, (C-terminal) antibody (Sigma Aldrich, Cat#SAB1404621, clone 6F7)
https://www.sigmaaldrich.com/catalog/product/sigma/sab1404621?lang=en&region=US

PTGES2 Mouse Monoclonal Antibody [Clone ID: OTI2C3] (OriGene, Cat# TA505412, clone OTI2C3)
https://www.origene.com/catalog/antibodies/primary-antibodies/ta505412/ptges2-mouse-monoclonal-antibody-clone-id-oti2c3

Monoclonal Anti-α-Tubulin antibody (Sigma Aldrich, Cat#T6074, clone B-5-1-2)
https://www.sigmaaldrich.com/catalog/product/sigma/t6074?lang=en&region=US

Anti-GAPDH antibody, Mouse monoclonal (Sigma Aldrich, Cat#G8795, clone GAPDH-71.1)
https://www.sigmaaldrich.com/catalog/product/sigma/g8795?lang=en&region=US

Monoclonal Anti-Actin antibody (Sigma Aldrich, Cat#A4700, clone AC-40)
https://www.sigmaaldrich.com/catalog/product/sigma/a4700?lang=en&region=US

Anti-biotin (D5A7) Rabbit mAb (Cell Signaling Technology, Cat#5597, clone D5A7)
https://www.cellsignal.com/products/secondary-antibodies/anti-biotin-d5a7-rabbit-mab/5597

IKKα Antibody (Cell Signaling Technology, Cat#2682S, polyclonal)
https://www.cellsignal.com/products/primary-antibodies/ikka-antibody/2682

RelB (D7D7W) Rabbit mAb (Cell Signaling Technology, Cat#10544, clone D7D7W)
https://www.cellsignal.com/products/primary-antibodies/relb-d7d7w-rabbit-mab/10544
c-Rel Monoclonal Antibody (1RELAH5) (ThermoFisher Scientific, Cat# 14-6111-82, clone 1RELAH5)
https://www.thermofisher.com/antibody/product/c-Rel-Antibody-clone-1RELAH5-Monoclonal/14-6111-82

ANTIBODIES USED IN VIVO

InVivoMAb anti-mouse IL-17A (Bio-X-Cell, Cat#BE0173, clone I7F3)
https://bxcell.com/product/m-il-17a/

InVivoMAb mouse IgG1 isotype control (Bio-X-Cell, Cat#BE0083, clone MOPC-21)
https://bxcell.com/product/mouse-igg1-isotype-control/

Validation

All antibodies have been verified by the supplying companies. Please see website URLs in the above section for more details.

Eukaryotic cell lines

Policy information about cell lines

Cell line source(s)	Cell lines RAW264.7 and CMT-93 cells were obtained from the American Type Culture Collection (ATCC).
Authentication	Cell lines were authenticated by the ATCC using Short Tandem Repeat (STR) analysis.
Mycoplasma contamination	Cell lines tested negative for mycoplasma contamination.
Commonly misidentified lines (See ICLAC register)	No commonly misidentified cell lines were used.

Animals and other organisms

Policy information about studies involving animals; ARRIVE guidelines recommended for reporting animal research

Laboratory animals	Laboratory mice (Mus musculus) were used in these experiments. C57BL/6 WT (Stock number 000664), Apc-Min (Stock number 002020), miR-146a-/- (Stock number 016239), miR-146afl/fl (Stock number 36799), LysM-Cre (Stock number 0004781), Villin-Cre (Stock number 0004586), and IL-17-GFP (Stock number 018472) mice were purchased from Jackson Laboratory. Myeloid-miR-146a-/- (Myeloid cell-miR-146a conditional knockout) mice were generated by crossing miR-146afl/fl with LysM-Cre mice. IEC-miR-146a-/- (intestinal epithelial cell-miR-146a conditional knockout) mice were generated by crossing miR-146afl/fl with Villin-Cre mice. All mice were age- (6-10 weeks old at the start of experiments) and sex-matched. Littermate controls were used where appropriate. Both male and female mice were used in the study. Mice were maintained in specific pathogen-free animal facilities at the Harvard Institutes of Medicine at Harvard Medical School and the Hale Building for Transformative Medicine at Brigham and Women's Hospital (Boston, MA). Mice were maintained at 20-25°C, 50-70% humidity, and a 12-hour light cycle with light phase beginning at 7 a.m. and ending at 7 p.m. Mice were housed with food and water ad libitum. All experiments were in accordance with guidelines from the Institutional Animal Care and Use Committee at Brigham and Women's Hospital.
Wild animals	No wild animals were used in this study.
Field-collected samples	No field-collected samples were used in this study.
Ethics oversight	All experiments were in accordance with guidelines from the Institutional Animal Care and Use Committee (Protocol 2016N000230) at Brigham and Women's Hospital.

Note that full information on the approval of the study protocol must also be provided in the manuscript.

Flow Cytometry

Plots

Confirm that:

☒ The axis labels state the marker and fluorochrome used (e.g. CD4-FITC).

☒ The axis scales are clearly visible. Include numbers along axes only for bottom left plot of group (a 'group' is an analysis of identical markers).

☒ All plots are contour plots with outliers or pseudocolor plots.

☒ A numerical value for number of cells or percentage (with statistics) is provided.

Methodology

Sample preparation	Murine bone marrow-derived dendritic cells and macrophages were prepared as described in the Methods section. Dendritic cells, macrophages, CD4+ alpha beta T cells, gamma delta T cells, and innate lymphoid cells were isolated from murine colonic lamina propria samples as described in the Methods section. Murine CMT-93 cell lines were prepared as described in the Methods section. Intracellular, surface, and live/dead staining were performed according to manufacturer's instructions.
Instrument	Model: FACS ARIA IIIu (Standard). 4-laser system (Blue 488nm, Red 640 nm, Yellow Green 561 nm, and Violet 405 nm).<br>Model: FACS ARIA IIu Special Order System (SORP). 3-laser system (Blue 488nm, Red 640 nm, and UV 350 nm).<br>LSR Fortessa X20 SORP. 5-laser system (Blue 488nm, Red 640 nm, Yellow Green 561 nm, UV 350 nm, and Violet 405 nm).<br>LSR II SORP. 3-laster system (Blue 488nm, Red 640 nm, and Violet 405 nm).<br><br>All are manufactured by BD (Beckton Dickinson) and all use the FACSDiva v8.0.2 interface.
Software	BD (Becton Dickinson) Biosciences FACSDiva v8.0.2 was used to acquire flow cytometry data.<br>BD (Becton Dickinson) Flowjo vs 10.7.1 was used to analyze flow cytometry data and prepare figures.
Cell population abundance	All populations were sorted with >95% purity. Please see figure panels for specific population frequencies.
Gating strategy	Please see figure legends for specific gating strategies. In general, viable cells were first gated broadly by size and internal complexity using forward scatter (FSC-A) and side scatter (SSC-A). We next gated on singlets and excluded doublets based on

cell size using FSC-H and FSC-W. Live cells were then gated using a viability dye (e.g. 7AAD- or fixable LIVE/DEAD-). Subsequent positive and negative gates were drawn based on established population boundaries with the assistance of appropriate fluorescence minus one (FMO) controls.

☒ Tick this box to confirm that a figure exemplifying the gating strategy is provided in the Supplementary Information.
